# *Ceratonia siliqua* pod extract ameliorates *Schistosoma mansoni*-induced liver fibrosis and oxidative stress

**DOI:** 10.1186/s12906-016-1389-1

**Published:** 2016-11-08

**Authors:** Ebtesam M. Al-Olayan, Manal F. El-Khadragy, Reem A. Alajmi, Mohamed S. Othman, Amira A. Bauomy, Shaimaa R. Ibrahim, Ahmed E. Abdel Moneim

**Affiliations:** 1 Department of Zoology, Faculty of Science, King Saud University, Riyadh, Kingdom of Saudi Arabia; 2 Department of Zoology and Entomology, Faculty of Science, University of Helwan, Cairo, Egypt; 3 Faculty of Preparatory year, University of Hail, Hail, Kingdom of Saudi Arabia; 4 Faculty of Biotechnology, October University for Modern Science and Arts (MSA), Giza, Egypt; 5 Laboratory Sciences Department, College of Science and Arts, Qassim University, Al-Rass, Kingdom of Saudi Arabia; 6 Molecular Drug Evaluation Department, National Organization for Drug Control & Research (NODCAR), Giza, Egypt

**Keywords:** *Ceratonia siliqua*, *Schistosoma mansoni*, Liver fibrosis, TIMP-2, Oxidative stress

## Abstract

**Background:**

Schistosomiasis is a prevalent parasitic disease found predominantly in tropical and sub-tropical areas of the developing world, with the second highest socioeconomic and public health burden despite strenuous control efforts. In the present study, we aimed to investigate the ameliorative effects of *Ceratonia siliqua* pod extract (CPE) on liver fibrosis and oxidative stress in mice infected with *Schistosoma mansoni*.

**Methods:**

The schistosomal hepatopathologic mouse model was established by tail immersion with schistosomal cercaria. The extract was given daily for 10 days beginning 42 days post-infection. Liver samples were obtained from mice sacrificed 9 weeks after infection. Liver histopathological changes were observed with hematoxylin-eosin and Masson trichrome staining.

**Results:**

Typical schistosomal hepatopathologic changes were induced in the untreated mice. However, the oral administration of CPE was effective in reducing worm number and the egg load in the liver. This treatment also decreased granuloma size and collagen deposition by inhibiting tissue inhibitor of metalloproteinases-2 (TIMP-2) expression. Schistosomal infection induced oxidative stress by increasing lipid peroxidation (LPO) and nitrite/nitrate (nitric oxide; NO) production along with concomitant decreases in glutathione (GSH) and various antioxidant enzymes, including superoxide dismutase, catalase, glutathione peroxidase and glutathione reductase. However, treatment of mice with CPE at 300 or 600 mg/kg inhibited LPO and NO production, increased GSH content, and restored the activities of the antioxidant enzymes compared with untreated infected mice. Furthermore, treatment with CPE inhibited apoptosis, as indicated by the reduced Bax expression in hepatic tissue.

**Conclusion:**

These data indicated that extracts from *Ceratonia siliqua* pods may play an important role in combating schistosomal hepatopathology and may inhibit granuloma formation and liver fibrosis through down-regulation of TIMP-2 expression.

**Electronic supplementary material:**

The online version of this article (doi:10.1186/s12906-016-1389-1) contains supplementary material, which is available to authorized users.

## Background

Human schistosomiasis is caused by parasitic helminths of the genus Schistosoma and is considered, after malaria, to be one of the most prevalent and debilitating neglected diseases in tropical and subtropical areas [1]. There are six main species of Schistosoma that can infect humans: *S. mansoni*, *S. hematobium*, *S. japonicum*, *S. intercalatum*, *S. malayensis* and *S. mekongi*. Among these, only *S. mansoni* and *S. hematobium* are found in Egypt [2]. Most of the pathology in *Schistosoma*-infected humans is attributed to the host’s reaction to the eggs, which is maximal by the 8th week of infection. The toxic egg material destroys cells in host tissues, and the antigenic material stimulates the development of large inflammatory reactions (granulomas) around the egg [3]. Such granulomas are considered protective barriers that sequester the toxic and antigenic substances secreted continuously by *Schistosoma* eggs. Moreover, the laid eggs stimulate oxidative processes and, elevate lipid peroxidation by impairing antioxidant defense mechanisms and causing peroxidative damage to lipid in liver cell membranes [4].

Fibrosis involves the excessive deposition of collagen and other extracellular matrix (ECM) components [5]. Complete recovery from liver fibrosis involves remodeling and breakdown of multiple ECM components. Degradation of the predominant component, collagen I, is regulated by the matrix metalloproteinase (MMP) family, which consists of calcium-dependent enzymes that specifically degrade collagenous and non-collagenous substrates [6]. However, there is increasing evidence increased expression of endogenous MMP inhibitors, i.e., tissue inhibitor of metalloproteinases (TIMP) family members, in fibrotic liver tissue may inhibit collagenase activity. The expression levels of both TIMP-1 and TIMP-2 were found to be elevated in human and animal models of liver fibrosis [7, 8]. The resulting increase in TIMP activity in liver tissues may promote fibrosis by protecting the deposited ECM from degradation by MMPs [6].

The treatment for schistosomiasis is based on the use of praziquantel (PZQ), a low-cost anthelmintic that is highly effective against all Schistosoma species that infect humans. Despite its benefits, PZQ induces considerable adverse clinical effects within 24 h after treatment. The mechanism of the side-effects of short-term PZQ treatment has not been clarified [9]. As a result, low cure rates and treatment failure following PZQ administration together with the existence of resistant strains, reinforce the need to develop new safe and effective schistosomicidal drugs.

The carob tree (*Ceratonia siliqua* L., Leguminosae family) is widely cultivated in the Mediterranean area. An important ingredient in carob seeds is galactomannan, which is known for its thickening effects and is widely used in the food industry. The crude extract of the plant pod exhibits antioxidant properties due to the presence of catechin, epicatechin, epigallocatechin, epigallocatechin gallate, and epicatechin gallate, along with simpler phenolics, such as phloroglucinol, pyrogallol, catechol, and gallic acid [10–12]. Avallone et al. [13] revealed that an extract of carob pods showed anxiolytic-sedative effects and acted as a chemopreventive agent. This extract also has antidepressant effects [14]. Dietary fiber from carob pods has also been shown to reduce total cholesterol and LDL-c levels [15]. Furthermore, carob pods extract can exert antiproliferative effects in vitro [16], with potential antitumor activity.

An in vivo study in small ruminants showed that *Ceratonia siliqua* and other Mediterranean plants had bioactive properties and could potentially be used as nutraceuticals against gastrointestinal parasitic nematodes [17]. Further, Arroyo-Lopez et al. [18] recently found that feeding lambs with tanniniferous resources from *Ceratonia siliqua* had a positive effect against gastrointestinal parasitic nematodes by affecting certain biological traits of the worm populations, including eggs per gram of feces and worm numbers. Therefore, in this study, we investigated the antioxidant and possible anti-schistosomal effects of carob pod extract on *S. mansoni*-infected mice.

## Methods

### Chemicals and experimental animals

Nitro blue tetrazolium, N-(1–naphthyl) ethylenediamine and Tris–HCl were purchased from Sigma (St. Louis, MO, USA). Thiobarbituric acid and trichloroacetic acid were purchased from Merck. All other chemicals and reagents used in this study were of analytical grade. Double-distilled water was used as the solvent.

Forty eight CD-1 Swiss male albino mice, 20–25 g in weight, were provided by the Schistosome Biology Supply Center (SBSC) of the Theodor Bilharz Research Institute (TBRI), Giza, Egypt. The mice were maintained on a standard commercial pelleted diet in an air-conditioned animal house at 22–25 °C. Animals were bred under specified pathogen-free conditions and fed a standard diet and water *ad libitum*.

### Preparation of carob pod extract

Carob pods were collected from an open market in East Cairo, Egypt in February and March 2014. The plant material was authenticated in the Botany Department, Faculty of Science, Helwan University, Cairo, Egypt by Prof. Dr. L. Hassan at the Herbarium of Helwan University (herbarium number: 5431). The seeds were removed and the carob pods were ground to particles of ≤ 1 mm in size. The powder was sieved and kept in a deep freezer (−20 °C) until the time of use. One hundred grams of dry fine powder was extracted with 20× (w/v) hot water (85 °C) for 1 h, after then, the mixture was incubated at 4 °C for 48 h with mixing from time to time. The extract was filtered with Whatman No. 1 filter paper to remove insoluble particles. The filtrate was lyophilized with a freeze-dryer-cryodo. The dried extract was then stored at −20 °C until used and designated as CPE. The amounts of polyphenols and flavonoids compounds were determined in the CPE using standard methods.

### Qualitative phytochemical screening for secondary metabolites

Carob pods extract was phytochemically screened for the presence of secondary metabolites. Qualitative phytochemical analyses for the presence of alkaloids, tannins including gallic, catechol tannis and reducing sugars were carried out by standard protocols.

### HPLC analysis

Analysis of the carob pods was performed using a Perkin Elmer Series 200 liquid chromatography (PerkinElmer, USA). The HPLC system was equipped with an autosampler, a C18 column from Teknokroma (Barcelona, Spain) and a photo diode array detector (PDA; model Series 200) The mobile phase was composed of water:methanol:glacial acetic acid (65:34:1 v/v) with the gradient elution system at a flow rate of 0.8 ml/min. 25 μl was injected after filtration through a 0.22 μm PVDF membrane. The detection UV wavelength was set at 280 nm. The column temperature was 28 °C.

### Infection of mice

*Schistosoma mansoni* cercariae were obtained from the Schistosoma Biological Supply Center at TBRI. Mice were exposed to *S. mansoni* (70 ± 5 cercariae/mouse) using the tail immersion method as modified by Oliver and Stirewalt [19].

### Experimental design

Animals were allocated to five groups of seven mice each. Group I served as the normal control; these mice received water (100 μl water/mouse) by oral administration for 10 days. Groups II, III, IV and V were infected with *S. mansoni*. The animals in Group II served as the vehicle control: beginning on day 46 post-infection with *S. mansoni*, they received water (100 μl water/mouse) by oral administration for 10 days. Group III was treated with PZQ at 500 mg/kg bwt in 70 % glycerine on two successive days. Groups IV and V were orally gavaged with 100 μl of 300 and 600 mg/kg bwt carob pod extract, respectively, daily for 10 days. We decided to start the treatments on day 46 post-infection (PI) to allow for the clinical expression of the disease and to test the efficacy of the extract in attenuating the pathophysiological features of the disease.

Twenty-four hours following the last administration, on day 56 post-infection with *S. mansoni*, the animals were killed under mild ether anesthesia, and blood samples were collected for serum analysis. The liver was carefully removed and washed twice in ice-cold 50 mM Tris–HCl, pH 7.4. Each liver was then weighed and immediately homogenized to yield a 10 % (w/v) homogenate in ice-cold medium that contained 50 mM Tris–HCl, pH 7.4. The homogenates were centrifuged at 3000 rpm for 10 min at 4 °C. The supernatants were used for the various biochemical determinations. The total protein content of the homogenized liver was determined by the Lowry method [20] using bovine serum albumin as a standard.

### Liver perfusion

Worms were recovered from the hepatic portal system and liver by a perfusion technique described by Aly and Mantawy [21]. The worms from each mouse were left to sediment for approximately 20 min in a small Petri dish to allow sex identification, examination and counting. The degree of protection or the percentage reduction in challenge was calculated as *P* = C − T/C × 100, where P is the level of protection, C is the mean number of parasites recovered from untreated infected mice, and T is the mean number of the parasites recovered from treated mice.

### Liver function test

Colorimetric determination of serum alanine aminotransferase (ALT) and aspartate aminotransferase (AST), activity was performed by measuring the amount of pyruvate or oxaloacetate produced by the formation of −2, −4-dinitrophenylhydrazine, according to the method of Reitman and Frankel [22].

### Biochemical analysis and oxidative stress markers

Liver homogenates were subjected to a thiobarbituric acid reaction to determine lipid peroxidation (LPO) levels expressed in terms of the amount of malondialdehyde (MDA) formed [23]. Similarly, nitrite/nitrate (nitric oxide; NO) and glutathione (GSH) were assayed by the methods of Green et al. [24] and Ellman [25], respectively.

### Enzymatic antioxidant status

Liver homogenates were used for the determination of superoxide dismutase (SOD) according to Nishikimi et al. [26], catalase as described by Aebi [27], glutathione peroxidase (GPx) according to Paglia and Valentine [28] and glutathione reductase (GR) as described by Factor et al. [29].

### Histopathological examination

Pieces of liver tissue were collected from all mice, fixed in 10 % phosphate buffered formalin, trimmed, processed conventionally, embedded in paraffin, sectioned at 5 μm and stained with hematoxylin and eosin or Masson’s trichrome for histopathological evaluation and image analysis. Unstained sections of each series were used for immunohistochemical studies. The granuloma size (μm^2^) was defined by the area containing a single schistosome egg, and liver sections from 5 mice in each group were randomly chosen for statistical analysis of granuloma areas.

### Immunohistochemical analyses of TIMP-2 and Bax

The immunolocalization technique used for TIMP-2 and Bax was performed on 3 to 4 μm thick sections according to Pedrycz and Czerny [30]. For negative controls, the primary antibody was omitted. In brief, mouse anti-Bax or anti-TIMP-2 (diluted1:200, Santa Cruz Biotechnology, Santa Cruz, CA, USA) antibodies were incubated with sections for 60 min, diluted in TBS (Tris-buffered saline)/1 % BSA (bovine serum albumin). A biotinylated secondary antibody directed against mouse immunoglobulin (Biotinylated Link Universal–DakoCytomation kit, supplied ready to use) was then added and incubated for 15 min, followed by horse radish peroxidase conjugated with streptavidin (DakoCytomation kit, supplied ready to use) for an additional 15 min of incubation. 3-amino-9-ethylcarbasole (AEC) (DakoCytomation kit, supplied ready to use) was then applied for 15 min, yielding a reddish brown color at the sites of immunolocalization of the primary antibodies. Specimens were counterstained with hematoxylin for 1 min and mounted using Aquatex fluid (Merck KGaA, Germany). The staining intensity was graded as very weak, weak, medium, or strong. All sections were incubated under the same conditions and at the same time with the same concentration of antibodies to ensure that the immunostaining would be comparable among the different experimental groups.

### Molecular assay (real-time PCR)

Total RNA was isolated from the liver tissues using an RNeasy Plus Minikit (Qiagen, Valencia, CA). One microgram total RNA and random primers were used for cDNA synthesis with the Script™ cDNA synthesis kit (Bio-Rad, CA). For real-time PCR analysis, the cDNA samples were run in triplicate. Real-time PCR reactions were performed using Power SYBR® Green (Life Technologies, CA) on an Applied Biosystems 7500 Instrument. The typical thermal profile for the PCR reaction was 95 °C for 4 min, followed by 40 cycles of 94 °C for 60s and 55 °C for 60s. After PCR amplification, the ΔCt was calculated by subtracting the *Actb* Ct from each sample Ct. The PCR primers for the *Gpx1* and *Sod2* genes were synthesized by Jena Bioscience GmbH (Jena, Germany). Primers were designed using the Primer-Blast program from NCBI. The PCR primer sequences were BLAST searched to ensure specificity for the particular gene. *Actb* was used as a reference gene.

The primer pairs used included:

*Sod2* (Accession number: NM_001270850.1; S): 5’-AGCTGCACCACAGCAAGCAC-3’, (AS): 5’-TCCACCACCCTTAGGGCTCA-3’.

*Gpx1* (Accession number: NM_017006.2; S): 50-CGGTTTCCCGTGCAATCAGT-3’, (AS): 5’-ACACCGGGGACCAAATGATG-3’.

*Actb* (Accession number: NM_031144.3; S): 5’-GGCATCCTGACCCTGAAGTA-3’, (AS): 5’-GGGGTGTTGAAGGTCTCAAA-3’.

### Statistical analysis

The results are expressed as the mean ± standard error of the mean (SEM). One-way ANOVA was carried out, and the statistical comparisons among the groups were performed with Duncan’s test using a statistical package (SPSS version 17.0). All p values are two-tailed and *p* < 0.05 was considered as significant for all statistical analyses in this study.

## Results

**Fig. 1:**
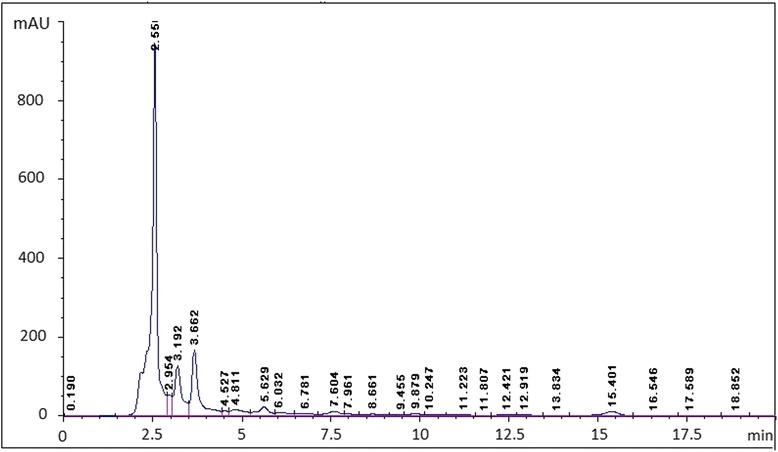
HPLC chromatogram of *Ceratonia siliqua* pod extract

**Fig. 2:**
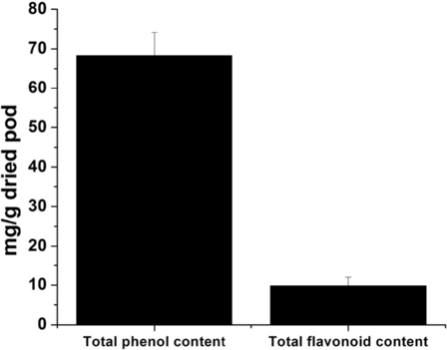
Total polyphenols and flavonoids contents in *Ceratonia siliqua* pod methanolic extract. The total polyphenols is determined as mg/g gallic acid equivalent of phenols/g dried pod whereas, the total flavonoid content is determined as mg/g quercetin equivalents of flavonoids/g dried pod. Data are represented as mean ± standard deviations of three independent experiments each performed in duplicate

The use of HPLC method (Fig. 1) revealed the identification of various phenolic compounds in *Ceratonia siliqua* pod, and as shown in the Additional file 1: Table S1, the principal compounds are: kaempferol (53.53 %), gallic acid (11.19 %), quercetin rhamnoside (8.32 %) polydatin (2.45 %), fraxidin (2.21 %) and cinnamic acid derivative (2.11 %). Furthermore, *Ceratonia siliqua* pod gave positive tests for alkaloids, catechol tannins and reducing sugars while it gave negative result for gallic tannins as shown in Table 1. The total polyphenols (TPC) and total flavonoids (TFC) contents present in the investigated CPE are shown in Fig. 2, which were found to be 68.3 ± 5.8 mg gallic acid equivalent/g dried pod of polyphenols. However, the amount of total flavonoids is relatively low and we found its amount is 9.8 ± 2.2 mg querestin equivalent/g dried pod. These results clearly suggest that the higher levels of antioxidant activity of this pod are due to the presence of polyphenols and flavonoids.

**Table 1:** Phytochemicals detected of alkaloids, different tannins and reducing sugars in *Ceratonia siliqua* pod

Parameter	*Ceratonia siliqua* pod
Alkaloids	+
Gallic tannins	−
Catechol tannins	+
Reducing sugars	+

Key: + = present; − = absent

Table 2 shows the worm burden and ova count in infected and treated mice. Treatment of infected mice with 300 or 600 mg/kg CPE significantly reduced the number of *S. mansoni* worms recovered from infected mice by 39.0 and 54.2 %, respectively (*p* < 0.05). In addition, the count of ova/g liver tissue in CPE-treated infected mice was reduced by 50 and 72.8 %, respectively (*p* < 0.05). Following CPE (300 and 600 mg/kg) treatment starting at day 46 post-infection, the oogram pattern revealed a complete disappearance of all immature and mature ova (data not shown). At the same time, administration of PZQ at day 46 post-infection caused the complete death of ova, as expected (Additional file 2).

**Table 2:** Effect of carob pods extract (CPE) administration on worm burden, liver tissue eggs load and granuloma size of *S. mansoni* infected mice

Groups	Mean number of worms ± SEM	Reduction on worm burden (%)	Ova count in liver ± SEM	Reduction on ova count (%)	Granuloma size (μm^2^)
Vehicle control	15.3 ± 1.37	-	11.4 ± 1.08	-	968500 ± 1364
PZQ (500 mg/kg bwt)	4.7 ± 0.52^a^	69.5^a^	2.3 ± 0.71^a^	79.8^a^	915400 ± 1519
CPE (300 mg/kg bwt)	9.3 ± 1.36^a^	39.0^a^	5.7 ± 0.34^a^	50.0^a^	882200 ± 757^a^
CPE (600 mg/kg bwt)	7.0 ± 0.89^a^	54.2^a^	3.1 ± 0.28^a^	72.8^a^	808900 ± 361^a^

Values are means ± SEM (*n* = 5). ^a^ *p* < 0.05, significant change with respect to the Vehicle control

**Fig. 3:**
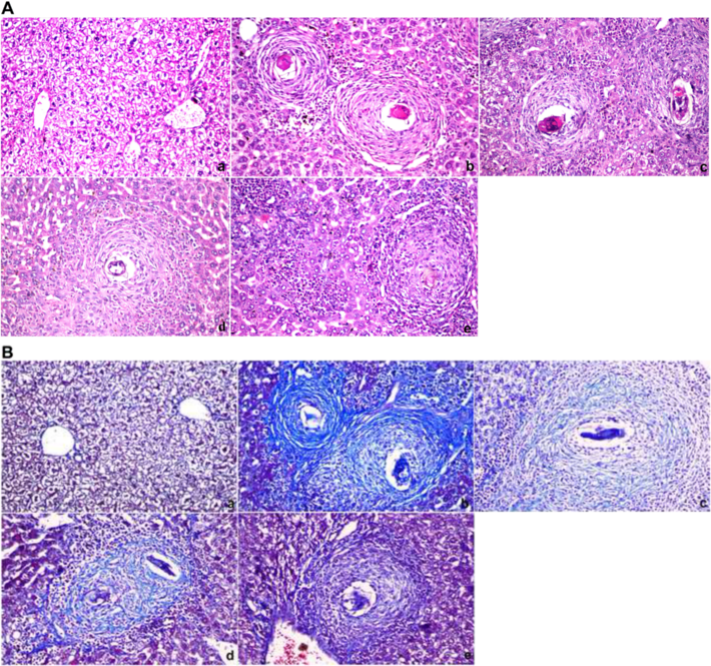
Photomicrographs of the liver 45 days post-infection stained with hematoxylin and eosin (**a**) or with Masson Trichrome (**b**). *a*: Control group, showing normal portal triad along with a normal hepatocytes and the central vein with the tyoical distribution of connective tissue. *b*: Vehicle control group, showing inflammatory cells, granulomatous lesions and focal areas of necrosis with condensed fibrous connective tissue. *c*: PZQ (500 mg/kg bwt)-treated group, showing smaller granulomatous lesions and condensed fibrous connective tissue. *d*: CPE (300 mg/kg bwt)-treated group, showing inflammatory cells around a central fibrous tissue granuloma with reduced formation of cellular epithelioid cells with central localized parasites and less fibrous tissues. *e*: CPE (600 mg/kg bwt)-treated group showing a massive number of leukocytes surrounding the granuloma area with less fibrous tissues (×400)

Histopathological studies confirmed the parasitological results (Fig. 3). The schistosomal liver showed both cellular and fibrous granuloma formation with central necrosis, condensed connective fibrous tissue, centrally localized embedded parasites and leukocyte infiltration at the periphery of the hepatic parenchyma. An obvious improvement was noticed in the CPE-treated groups, with reduced formation of cellular epithelioid cells and fewer centrally localized parasites with less condensed connective tissue. Decreases in granuloma size were also observed in CPE-treated groups (Table 2).

**Fig. 4:**
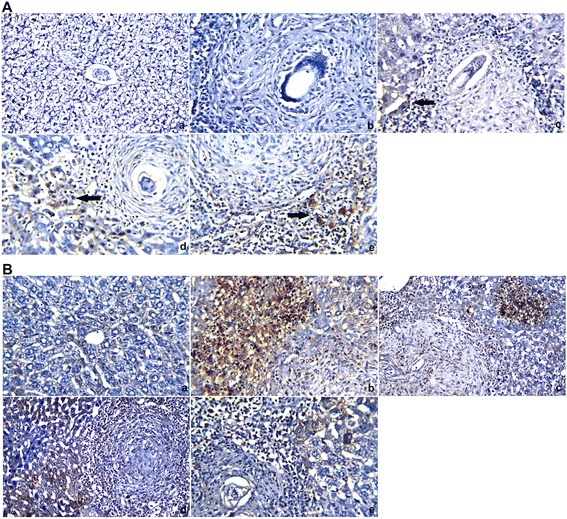
Mouse livers stained with TIMP-2 and Bax antibodies (**a** and **b**, respectively). *a*: Control group, with negative (−ve) TIMP-2 and Bax immunoreactivity in the hepatocytes and central vein. *b*: Vehicle control group, with negative (−ve) TIMP-2 immunoreactivity in the hepatocytes and strong positive (+ve) Bax immunoreactivity in hepatic tissues. *c*: PZQ (500 mg/kg bwt)-treated group, with mild TIMP-2 immunoreactivity and strong positive (+ve) Bax immunoreactivity in the hepatic tissues adjacent to the granuloma. *d*: CPE (300 mg/kg bwt)-treated group, showing positive (+ve) TIMP-2 immunoreactivity in the hepatic tissues surrounding the granuloma and mild Bax immunoreactivity in the hepatocytes adjacent to the fibrous granuloma. *e*: CPE (600 mg/kg bwt)-treated group, with positive (+ve) TIMP-2 immunoreactivity and weak Bax immunoreactivity in the hepatocytes adjacent to the fibrous granuloma (×400)

An immunohistochemical examination of control and untreated infected livers showed negative immunoreactivity for TIMP-2 in hepatocytes (Fig. 4). In the PZQ-treated group, mild TIMP-2 immunoreactivity was observed in the hepatic tissues adjacent to granulomas. CPE treatments induced positive TIMP-2 immunoreactivity in the hepatic tissues surrounding granulomas indicating that CPE has anti-fibrous activity of CPE. An immunohistochemical analysis of Bax expression revealed no immunoreactivity in the hepatic tissues of the control group but increased staining in the hepatic tissues of infected and PZQ-treated mice. By contrast, CPE treatments resulted in weak to mild Bax immunoreactivity in the hepatocytes adjacent to the fibrous granulomas in the liver.

As observed in Table 3, the infected but untreated mice (Group II) showed elevated activities of two enzymes that reflect hepatic functional parameters, namely ALT and AST. This elevation in hepatic functional parameters was also observed in the PZQ-treated mice. Conversely, these markers were maintained at near normal levels in the CPE (300 and 600 mg/kg) treatment groups.

**Table 3:** Serum ALT and AST of the studied groups

Groups	ALT (IU/L)	AST (IU/L)
Normal control	87.97 ± 6.8	77.40 ± 4.8
Vehicle control	118.86 ± 5.8^a^	108.66 ± 9.9^a^﻿
PZQ (500 mg/kg bwt)	105.75 ± 5.6^a^	107.55 ± 8.9^a^
CPE (300 mg/kg bwt)	86.54 ± 7.5^ab^	87.64 ± 7.9^ab^
CPE (600 mg/kg bwt)	81.30 ± 5.9^ab^	81.65 ± 8.4^ab^

Values are means ± SEM (*n* = 7)

^a^ *p* < 0.05, significant change with respect to Control group (Group I); ^b^ *p* < 0.05, significant change with respect to Vehicle group (Group II) for Duncan’s post hoc test

**Fig. 5:**
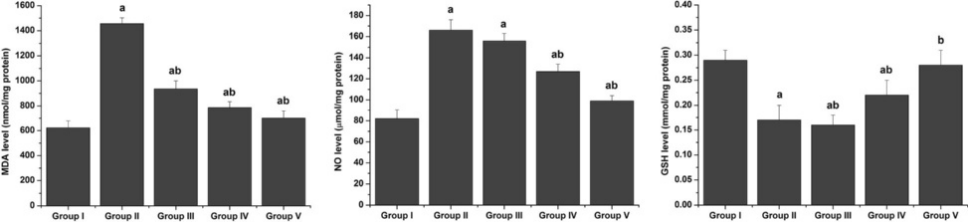
Effect of CPE and PZQ on oxidative stress markers. Values are the mean ± SEM (*n* = 7). ^a^ *p* < 0.05, significant change with respect to the Control group; ^b^ *p* < 0.05, significant change with respect to the Vehicle control group. LPO: lipid peroxidation; NO: nitric oxide; GSH: glutathione

To examine the effect of *S. mansoni* infection on oxidative stress markers, lipid peroxidation and nitric oxide levels were measured in hepatic homogenates (Fig. 5). Schistosomiasis caused a significant (p˂0.05) increase in the liver levels of LPO and NO compared with the control group. CPE treatments significantly (*p* < 0.05) reversed this elevation in LPO and NO levels. Schistosomiasis also induced hepatic oxidative stress, indicated by a significant reduction (*p* < 0.05) in the GSH contents of liver tissues from untreated infected mice compared with the control group. This reduction in GSH contents was attenuated by post-infection treatment with CPE (Fig. 5).

**Fig. 6:**
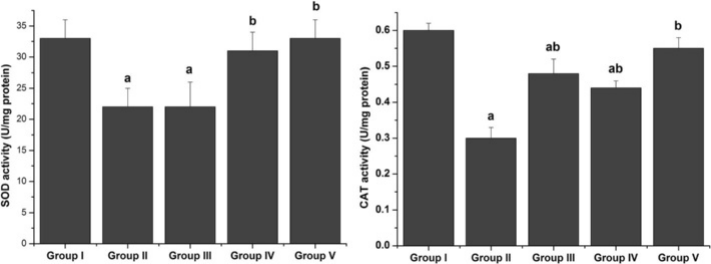
Effect of CPE and PZQ on hepatic antioxidant enzyme activities. Values are the mean ± SEM (*n* = 7). ^a^ *p* < 0.05, significant change with respect to the Control group; ^b^ *p* < 0.05, significant change with respect to the Vehicle control group. SOD: superoxide dismutase; CAT: catalase

**Fig. 7:**
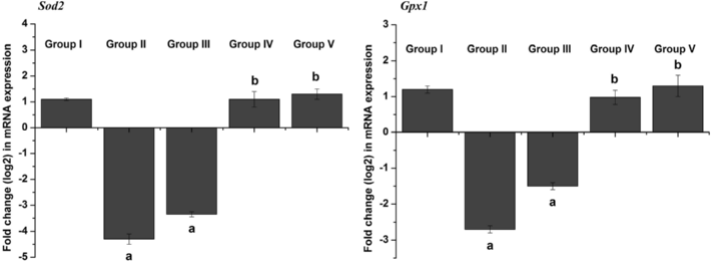
Effect of CPE and PZQ on the hepatic mRNA expression of candidate genes. Results (mean ± SEM of three assays) were normalized to the *Actb* mRNA level and are shown as fold induction (log2 scale) relative to the mRNA level in the control. *Sod2*: superoxide dismutase; *Gpx1*: glutathione peroxidase 1

To examine how schistosomiasis induced oxidative stress in liver tissue, potential modulations in the antioxidant defense system were investigated, with a focus on the activities of SOD, CAT, GST, GPx and GR enzymes. As shown in Fig. 6 and Table 4, *S. mansoni* infection led to changes in the activities of antioxidant enzymes relative to control mice. After 8 weeks of *S. mansoni* infection, SOD, CAT, GST, GPx and GR activities in liver homogenates were significantly decreased (*p* < 0.05) compared with controls. This suppression was significantly relieved by CPE treatment (*p* < 0.05). Real-time qPCR assays further showed that *Sod2* and *Gpx1* gene expression was reduced in the livers of the *S. mansoni*-infected mice but restored in infected mice treated with CPE (Fig. 7).

**Table 4:** Liver antioxidant enzyme activities of the studied groups

Groups	GR (μmol/mg protein)	GPx (U/mg protein)	GST (U/mg protein)
Normal control	28.47 ± 5.2	685.45 ± 38.97	46.24 ± 3.05
Vehicle control	17.95 ± 3.86^a^	576.89 ± 47.96^a^	14.90 ± 2.87^a^
PZQ (500 mg/kg bwt)	19.76 ± 4.30^a^	580.44 ± 67.87^a^	25.76 ± 3.98^ab^
CPE (300 mg/kg bwt)	24.76 ± 3.76^b^	604.55 ± 44.77^ab^	36.85 ± 2.69^ab^
CPE (600 mg/kg bwt)	27.56 ± 3.64^b^	630.67 ± 22.87^b^	39.65 ± 3.68^b^

Values are means ± SEM (*n* = 7)

^a^ *p* < 0.05, significant change with respect to Control group (Group I); ^b^ *p* < 0.05, significant change with respect to Vehicle group (Group II) for Duncan’s post hoc test

## Discussion

Previous works have shown that the relationship between schistosoma and the host is extremely complex. An early event following liver injury by *S. mansoni* infection is the production of excessive reactive oxygen species (ROS) which induce hepatic stress [31]. In the present work, *S. mansoni* infection impaired the antioxidant system, as the level of GSH, the main endogenous antioxidant, was depleted in association with elevated lipid peroxidation, a marker of cellular oxidative stress that has long been recognized as a major consequence of oxidative damage in different diseases. MDA is the end product of lipid peroxidation. In the present study, elevated MDA levels were observed in the livers of infected mice. Lipid peroxidation has traditionally been considered the major effect of free radicals and lipid peroxidation products impair the physicochemical properties, fluidity and integrity of cell membranes and lead to damage and cell necrosis [32]. In this study, treatment of infected mice with CPE increased hepatic GSH contents potentially through the well established antioxidant actions of carob [33], which reduced the formation of intracellular ROS in response to different pro-oxidant stimuli. The present data suggest that carob can protect liver cells by preventing liver glutathione depletion and stabilizing membrane permeability by inhibiting the formation of MDA, a convenient biomarker for lipid peroxidation and the most mutagenic product of lipid peroxidation.

In the present study, the activities of the antioxidant enzymes SOD, CAT, GST, GPx and GR were also decreased in the liver tissues of mice infected with *S. mansoni*. CPE treatment of infected mice restored the activities and expression levels of these antioxidant enzymes. Therefore, treatment with CPE may protect hepatocytes from the damage, demise and dysfunction caused by *S. mansoni*-induced oxidative stress at the sites of infection [34].

The protective effects of CPE in maintaining GSH at near control levels should increase the capacity of the endogenous antioxidant defense system. CPE may function by increasing steady state levels of GSH and/or its rate of synthesis, thereby conferring enhanced protection against oxidative stress.

The present results on antioxidant enzyme activities are in accordance with several previous studies that found significant GPx and GR reductions in the livers of infected mice and attributed these changes to the numerous deleterious effects caused by the accumulation of superoxide radicals and hydrogen peroxide. These dramatic changes during the infectious state can also be explained by the *S. mansoni* eggs trapped in the host liver, which elicit a chain of oxidative processes that may be, at least in part, responsible for the pathology and progression of fibrosis associated with schistosomal infection [35, 36].

Over production of hepatic NO in response to parasitic infection can be considered one of the factors that is responsible for inducing oxidative stress and inflicting tissue injury [2]. Excess NO is involved in the production of peroxynitrite (ONOŌ), which is a reactive nitrogen species (RNS) that oxidizes cellular structures, causing lipid peroxidation, and impairs the function of certain enzymes. NO production is also positively associated with tissue fibrosis due to its induction of fibrogenic cytokines and increased collagen synthesis [37].

*S. mansoni* causes a rather silent infection in the host until the parasite has accomplished oviposition, 8 weeks after infection. Some eggs laid in mesenteric vessels are carried by the blood flow and become trapped inside the liver. When eggs are caught in the liver, they can no longer be eliminated but instead promote a granulomatous reaction that isolates the eggs from the surrounding parenchyma. This explains the positive correlation between liver tissue egg counts and septal as well as total fibrosis scores. As a result, collagen is deposited around the eggs by myofibroblasts [38].

The collagen turnover and ECM remodeling that occur during various physiological and pathological processes including tissue repair, wound healing, fibrosis, and tumor invasion are largely dependent on the regulation of MMP and TIMP activities [6]. In the present study, liver sections of *S. mansoni*-infected mice showed moderate expression of TIMP-2. However, the CPE-treated group showed only mild expression of TIMP-2, indicating that CPE treatment led to collagen degradation and a consequent decrease in granuloma and fibrotic areas. The reduction in collagen deposition may also be related to active carob components scavenging free radicals and inhibiting inflammatory pathways that induce oxidative stress and tissue fibrosis.

Determination of liver enzyme function, reflected by serum AST and ALT levels, is commonly used to assess the liver damage caused by schistosomal infection [39]. These enzymes are important markers of hepatocellular damage as affirmed by Al-Olayan et al. [32]. In the present study, schistosomal infection significantly elevated serum levels of AST and ALT. This may be due to necrosis, with increased leakage of these enzymes from hepatocytes to the blood and replacement of normal liver tissue with granuloma lesions around entrapped schistosome eggs. Our data are in accordance with those of other authors [31, 40] who reported decreased AST and ALT levels in liver homogenates of *S. mansoni*-infected mice with concomitant increases in serum transaminases. The increase in enzyme activity is attributed to the irritation of liver cells by toxins or metabolic products of growing worms, adult worms and eggs [41].

Our study demonstrated that oral administration of CPE to infected mice was effective at reducing worm burden and egg count compared with untreated infected mice, indicating its useful antischistosomal action. Some authors have demonstrated that the death of worms upon treatment with antischistosomal drugs resulted from metabolic disorders, mechanical destruction or muscular xcontraction of treated worms [42, 43]. Furthermore, antioxidant supplementation can support the immune response through antioxidative mechanisms and thereby reduce infectious morbidity and help protect the host from pathogens [44]. Ali [41] demonstrated the importance of antioxidants in the treatment of schistosomal infection and the reduction of worm load as well as ova count.

## Conclusion

In conclusion, this study has shown that an extract from *Ceratonia siliqua* pods had significant antioxidant and anti-granuloma activities and caused a significant reversal of the *S. mansoni*-induced oxidative injury in the liver. These effects may be attributed to the various different polyphenols and flavonoids present in the extract. Our results thus demonstrate for the first time that *Ceratonia siliqua* pod extract has promising anti-schistosomal properties.

## Additional files

Additional file 1: Table S1. Identification of phenolic compounds by HPLC technique in *Ceratonia siliqua* pod extract. (DOC 43 kb)
Additional file 2: Table S2. Effect of carob pods extract (CPE) administration on oogram pattern of S. mansoni infected mice. (DOC 32 kb)

## Abbreviations

ALT: Alanine aminotransferase
AST: Aspartate aminotransferase
CAT: Catalase
CPE: *Ceratonia siliqua* pod extract
ECM: Extracellular matrix
GPx: Glutathione peroxidase
GR: Glutathione reductase
GSH: Glutathione
LPO: Lipid peroxidation
MDA: Malondialdehyde
MMP: Matrix metalloproteinase
NO: Nitric oxide
PZQ: Praziquantel
RNS: Reactive nitrogen species
ROS: Reactive oxygen species
SOD: Superoxide dismutase
TIMP-2: Tissue inhibitor of metalloproteinases-2
