# Erratum to: *Ceratonia siliqua* pod extract ameliorates *Schistosoma mansoni*-induced liver fibrosis and oxidative stress

**DOI:** 10.1186/s12906-016-1502-5

**Published:** 2017-01-17

**Authors:** Ebtesam M. Al-Olayan, Manal F. El-Khadragy, Reem A. Alajmi, Mohamed S. Othman, Amira A. Bauomy, Shaimaa R. Ibrahim, Ahmed E. Abdel Moneim

**Affiliations:** 1 Chair vaccines research of infectious diseases, Faculty of Science, King Saud University, Riyadh, Kingdom of Saudi Arabia; 2 Department of Zoology, Faculty of Science, King Saud University, Riyadh, Kingdom of Saudi Arabia; 3 Department of Zoology and Entomology, Faculty of Science, University of Helwan, Cairo, Egypt; 4 Faculty of Preparatory year, University of Hail, Hail, Kingdom of Saudi Arabia; 5 Faculty of Biotechnology, October University for Modern Science and Arts (MSA), Giza, Egypt; 6 Laboratory Sciences Dept., College of Science and Arts, Qassim University, Al-Rass, Kingdom of Saudi Arabia; 7 Molecular Drug Evaluation Department, National Organization for Drug Control & Research (NODCAR), Giza, Egypt

## Erratum

Following publication of the original article [1] it was brought to our attention that the affiliations for this article had been incorrectly presented. Please note that the correct affiliations should be as follows:

Ebtesam M. Al-Olayan^1,2^, Manal F. El-Khadragy^1,2,3^, Reem A. Alajmi^1^, Mohamed S. Othman^4,5^, Amira A. Bauomy^3,6^, Shaimaa R. Ibrahim^7^ and Ahmed E. Abdel Moneim^3*^

^1^Chair vaccines research of infectious diseases, Faculty of Science, King Saud University, Riyadh, and KSA

^2^Department of Zoology, Faculty of Science, King Saud University, Riyadh, KSA

^3^Department of Zoology and Entomology, Faculty of Science, University of Helwan, Cairo, Egypt

^4^Faculty of Preparatory year, University of Hail, Hail, KSA

^5^Faculty of Biotechnology, October University for Modern Science and Arts (MSA), Giza, Egypt

^6^Laboratory Sciences Dept., College of Science and Arts, Qassim University, Al-Rass, KSA

^7^Molecular Drug Evaluation Department, National Organization for Drug Control & Research (NODCAR), Giza, Egypt

Please also note that the Acknowledgements section should be updated to the following: “The authors extend their appreciation to the Deanship of Scientific Research at King Saud University for funding the work through the research group project No. RGPVPP-074”.
