# Supplementary material for: Ceratonia siliqua pod extract ameliorates Schistosoma mansoni-induced liver fibrosis and oxidative stress
Source: BMC Complement Altern Med. 2016 Nov 8;16:434. doi: 10.1186/s12906-016-1389-1 (PMC5100080; doi:10.1186/s12906-016-1389-1)
Supplement: Additional file 1: Table S1. — Identification of phenolic compounds by HPLC technique in Ceratonia siliqua pod extract. (DOC 43 kb) [file 12906_2016_1389_MOESM1_ESM.doc]

### Supplementary data SI: Identification of phenolic compounds by HPLC technique in *Ceratonia siliqua* pod extract.

| **Peak**  **#** | **Phenolic compounds** | **RT** | **Relative abundance (%)** |
| --- | --- | --- | --- |
|  | Kaempferol | 2.56 | 53.53 |
|  | Cinnamic acid derivative | 2.96 | 2.11 |
|  | Quercetin rhamnoside | 3.19 | 8.32 |
|  | Gallic acid | 3.66 | 11.19 |
|  | Gallic acid derivative | 4.53 | 0.67 |
|  | Fraxidin | 4.81 | 2.21 |
|  | Polydatin | 5.63 | 2.45 |
|  | Daidzein | 6.03 | 1.10 |
|  | Apigenin | 6.78 | 0.94 |
|  | 1,6-Di-O-galloyl-glucose | 7.60 | 1.54 |
|  | 1,2,6-Tri-O-galloyl-glucose | 7.96 | 0.55 |
|  | Myricetin glucoside | 8.66 | 0.88 |
|  | 1,2,3,6-Tera-O-galloyl-glucose | 9.46 | 0.33 |
|  | Myricetin rhamnoside | 9.88 | 0.76 |
|  | Syringic acid | 10.25 | 0.49 |

Abbreviation: RT, retention time.
