# Supplementary material for: Ceratonia siliqua pod extract ameliorates Schistosoma mansoni-induced liver fibrosis and oxidative stress
Source: BMC Complement Altern Med. 2016 Nov 8;16:434. doi: 10.1186/s12906-016-1389-1 (PMC5100080; doi:10.1186/s12906-016-1389-1)
Supplement: Additional file 2: Table S2. — Effect of carob pods extract (CPE) administration on oogram pattern of S. mansoni infected mice. (DOC 32 kb) [file 12906_2016_1389_MOESM2_ESM.doc]

**Supplementary data SII:** Effect of carob pods extract (CPE) administration on oogram pattern of *S*. *mansoni* infected mice.

| **Groups** | **Oogram pattern (%ova)** | | |
| --- | --- | --- | --- |
|  | **Immature** | **Mature** | **Dead** |
| **Vehicle control** | 65.0±3.8 | 31.3±3.5 | 3.7±2.2 |
| **PZQ** (500 mg/kg bwt) | 0.0±0.0^a^ | 0.0±0.0^a^ | 100.0±0.0^a^ |
| **CPE** (300 mg/kg bwt) | 35.2±4.7^a^ | 2.4±1.4^a^ | 62.4±5.7^a^ |
| **CPE** (600 mg/kg bwt) | 0.0±0.0^a^ | 0.0±0.0^a^ | 100.0±0.0^a^ |

Values are means ± SEM (n=5). ^a^*p*<0.05, significant change with respect to **Vehicle control**.
